# Feelings of Entrapment during the COVID-19 Pandemic Based on ACE Star Model: A Concept Analysis

**DOI:** 10.3390/healthcare9101305

**Published:** 2021-09-30

**Authors:** Hyun-Jung Lee, Bom-Mi Park

**Affiliations:** 1 Department of Nursing, The Catholic University of Korea, Seoul ST. Mary’s Hospital, Seoul 06591, Korea; leehj8505@korea.ac.kr; 2 Department of Nursing, Konkuk University, Chungju-si 27478, Korea

**Keywords:** feelings of entrapment, COVID-19 pandemic, academic center for evidence-based practice (ACE) star model, concept analysis, lockdown system, out of control, no escape, trapped, suicide, mental health

## Abstract

This study aimed to analyze the concept of the “feelings of entrapment” during the COVID-19 (coronavirus disease 2019) pandemic using a systematic review. We included literature based on content and outcomes related to feelings of entrapment, such as antecedents, attributes, and consequences. The exclusion criteria were studies that did not have inappropriate subject, content, conceptual definition, and degree thesis was excluded. Walker and Avant’s process of concept analysis was used in this systematic literature review. The attributes of the concept of feelings of entrapment during the COVID-19 pandemic were found to be feelings of: (1) being out of control, (2) no escape, (3) being trapped, (4) being robbed, and (5) hopelessness. The causes for these were identified as (1) the COVID-19 pandemic, (2) lockdown system, (3) restricted situation, (4) uncertain future, (5) economic hardship, and (6) poor coping abilities. Consequences of the concept were: (1) increased suicide, (2) decreased mental health, and (3) decreased well-being. In situations such as COVID-19, it is important need to know what feelings of entrapment’s antecedents and attributes are to prevent suicide and enhance mental health and well-being. Based on the results of this study, counseling services, policies, and systems for relieving feelings of entrapment in the COVID-19 situation are recommended.

## 1. Introduction

### 1.1. Necessity of The Study

The effects of COVID-19 (coronavirus disease 2019) on mental health and well-being are likely to be profound and long-lasting [1,2] and extend beyond those directly affected by the virus. However, it is unclear who will be affected and to what extent such effects will generalize across all aspects of mental health [3]. Many countries have adopted quarantine policies such as closing schools, factories, and other public places to slow the spread of COVID-19. The government has encouraged people to stay home and maintain social distancing [4]. However, coercive measures such as social distancing, lockdown, and travel restrictions due to COVID-19 have brought about many stressors, including unemployment, destitution, loss of business, domestic violence, and child abuse; primarily relating to people’s movement and interaction [5]. In addition, continuous social distancing can have a detrimental effect by making one feel isolated and excluded from society [6]. The World Health Organization reports that many suicides occur in crises. Identified risk factors for suicide, including loneliness, discrimination, financial problems, and mental health problems [7], are exacerbated during epidemics and disasters. In particular, the COVID-19 pandemic has a substantial impact on the mental health of the general population [1,8] and is associated with increased suicide cases [9]. Moreover, irresponsible reports of suicide surges have also instilled fear in people’s minds [10].

The main adverse effects of the COVID-19 pandemic include increased social isolation and loneliness closely related to anxiety, depression, self-harm, and suicide attempts [11]. According to the IMV-Model, entrapment is a core component of the psychological mechanisms underlying suicide ideation [12]

Gilbert and Allan (1998) differentiated between internal and external entrapment [13]. In a sustained stressful situation, the feeling of entrapment perceived as the motivation to escape from one’s inner feelings and thoughts is called internal entrapment [13]. In contrast, the sense of entrapment perceived as an escape motive from the surrounding external world is called external entrapment [13].

Most studies about entrapment and COVID-19 have dealt with suicides during COVID-19 [9,14], depression and anxiety [15], self-harm [16], and primarily report negative studies. However, Tesimann and Brailovkaia (2020) note said that suicide ideation is not an inevitable consequence of feelings of entrapment [17]. Amongst others, positive future thoughts, resilience, and social support are said to be “motivational moderators as they are thought to buffer against the emergence of suicidal ideation and intent” [12].

For individuals with low social cohesion during the COVID-19 pandemic, long-term isolation measures such as containment may lead to feelings of entrapment and suicide. However, these suicidal behaviors are subject to biopsychosocial effects [18]. For example, anti-inflammatory or related psychosocial therapy and media information may influence suicidal behavior, but a structured approach may be followed to address all possible causes of suicide adequately [18]. This understanding is essential to ensure that we are well prepared for potential further outbreaks of the COVID-19 pandemic. As the social and economic consequences of the lockdown and COVID-19 are likely to be associated with loneliness, social isolation, and pitfalls [3], we consider among these factors the psychological mechanisms underlying suicidal ideation in the Integrated Motivational-Volitional (IMV) Model. The core component of entrapment needs to be clearly defined [17]. By finding the antecedent factors for the feeling of entrapment, the positive factors can be improved. Similarly, by reducing the negative factors, the feeling of entrapment can be lowered, and ultimately the suicide rate.

The academic center for evidence-based practice (ACE) star model provides a comprehensive yet straightforward approach to putting evidence into practice [19]. In particular, it is possible to highlight the barriers encountered during this, specify solutions based on evidence-based practices (EBP) [20], and consider the knowledge transformation characteristics required for usefulness and relevance in clinical decision-making [20]. However, while nursing requires comprehensive knowledge and skills with a positive attitude toward EBP, there are still barriers to using it in the field [20]. Thus, by applying the ACE star model in this study, the concept of entrapment and its rise in the context of COVID-19 can be redefined, providing a new form of knowledge through the EBP process [20]. In addition, we can enhance healthcare availability and quality by steadily assessing the feelings of entrapment for the COVID-19 situation.

Good mental health and psychological well-being moderate the association between entrapment and suicide ideation. In addition, there is a need to identify both negative and positive psychological factors concerning the risk of suicidal ideation [17]. A conceptual analysis study on feelings of entrapment has yet to be conducted from the nursing perspective.

The concept of coercion in the era of COVID-19 has various connotations. Concept analysis is essential for academic development because it improves communication between concept users by finding precise meanings [21]. However, if the researcher do not correctly recognize it, the reliability and validity of the research may be threatened. Improving the quality of nursing care and patient outcomes are critical issues in current medical care [21]. Therefore, it is necessary to analyze this concept to define the feelings of entrapment during the COVID-19 pandemic distinctly.

In this study, an attempt was made to advance the nursing discipline with an improved understanding of entrapment feelings and provide essential data to healthcare workers and subjects of interest. Moreover, it is to use as basic data for the development of an intervention program can be used to reduce the feelings of entrapment in the future by identifying the antecedents, attributes, and consequences of the feelings of entrapment in the COVID-19 pandemic situation.

### 1.2. Purpose

The purpose of this study is to clarify the concept of feelings of entrapment and identify its attributes for a more precise understanding regarding its meaning, antecedents, and consequences.

## 2. Materials and Methods

### 2.1. Study Design

This systematic literature review uses a concept analysis that derives the attributes, antecedents, and consequences of feelings of entrapment during the COVID-19 pandemic using the method developed by Walker and Avant [22].

### 2.2. Data Selection Criteria

This study was conducted in accordance with the systematic literature review, following the Preferred Reporting Items for Systematic Review and Meta-Analysis (PRISMA) statement [23]. The inclusion criteria were studies that included literature including contents and outcomes related to feelings of entrapment, such as antecedents, attributes, and consequences. Since this study is a concept analysis of feelings of entrapment, participants, interventions, comparisons, and study designs were included without limitation. The exclusion criteria were studies that did not have inappropriate subject, content, conceptual definition, and degree thesis was excluded.

### 2.3. Study Details

We confirm the conceptual attributes and contextual referents by including the concept of “feelings of entrapment during the COVID-19 pandemic”. Searches were conducted using the keywords “feelings of entrapment”, “entrapment”, “entrapment and mental health”, “entrapment and psychological”, “entrapment and anxiety”, “entrapment and depression”, “entrapment and suicide”, and “COVID-19”.

**Figure 1:**
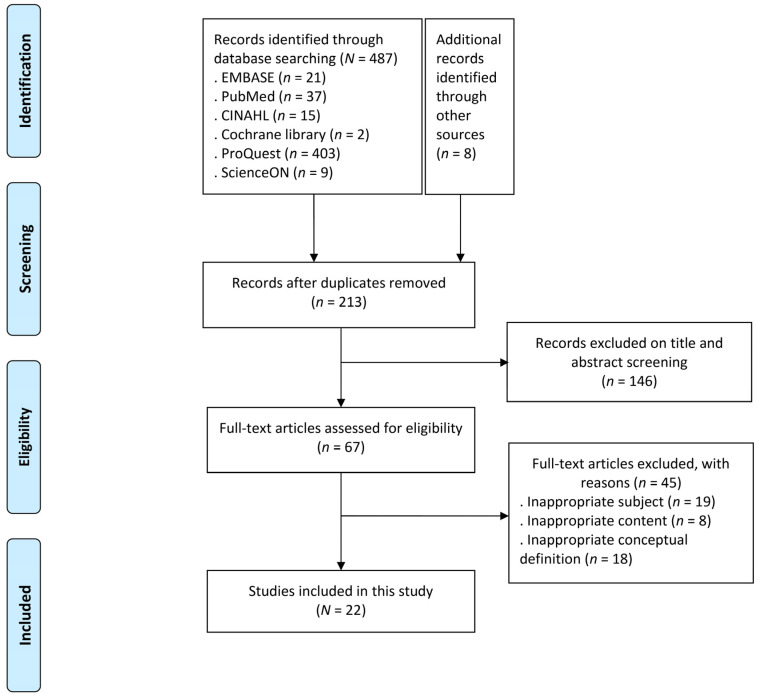
Flow diagram of study selection process.

We identify the conceptual attributes and confirm how the concept of feelings of entrapment was used and defined in the literature by conducting searches in the database (Embase, PubMed, CINAL, Cochrane, and ProQuest, ScienceON) published from 1 January 2020 to 31 July 2021. A total of 213 articles were extracted as primary sources using the keywords listed above. Of these, 67 secondary sources were extracted based on the title and abstract. Lastly, 22 articles were selected after excluding inappropriate content or conceptual definitions (Figure 1).

Content regarding feelings of entrapment during the COVID-19 pandemic was identified according to the protocol suggested by Walker and Avant [22]. The specific processes were as follows:

- Select the concept.
- Determine the purpose of the concept analysis.
- Identify the range of use of the concept.
- Identify the attributes of the concept.
- Present a model case of the concept.
- Present additional cases of the concept (borderline, contrary, and related cases).
- Identify antecedents and consequences of the concept.
- Define empirical referents of the concept.

### 2.4. Study Framework

The ACE star model is used for understanding the cycle, nature, and characteristics of knowledge utilized in various aspects of EBP [24]. It is a framework that can organize existing and new concepts and compose EBP processes and approaches. In addition, new information discovered at different stages of knowledge transformation can be transformed into practice, a general process that highlights unique aspects of EBP [24]. The ACE star model consists of five steps: (1) discovery research, (2) evidence summary, (3) translation to guidelines, (4) practice integration, and (5) process and outcome evaluation.

**Figure 2:**
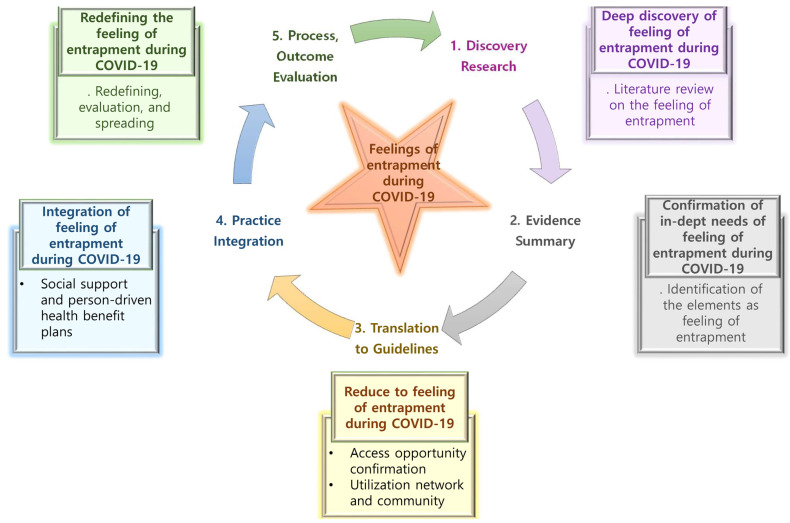
Conceptual framework of feelings of entrapment during the COVID-19 pandemic based on the ACE star model.

This study constructed the framework of feelings of entrapment based on the ACE star model (Figure 2). This includes:

(1) Discovery research: This involves knowledge generation, wherein new knowledge is discovered through historical literature.

(2) Evidence summary: This is the first inherent phase of the EBP, which gives knowledge a meaningful statement as a science. In this study, we identify the actual component of feelings of entrapment in a COVID-19 situation.

(3) Translation to guidelines: This is the process of converting and integrating EBP into practice. This study identifies access to understanding feelings of entrapment in a COVID-19 situation and provides an opportunity to reduce entrapment by utilizing different networks and communities for concepts.

(4) Practice integration: This is the process of changing both individual and organizational practices through formal and organized practices. This study provides social support and person-driven health benefit plans to reduce feelings of entrapment in a COVID-19 situation.

(5) Process and outcome evaluation: This evaluates the impact of EBP on health outcomes and leads to improvements in health care. In this study, the effects of redefining and evaluating feelings of entrapment in a COVID-19 situation can be considered to improve health care quality.

## 3. Results

### 3.1. Literature Review of Feelings of Entrapment

#### 3.1.1. Lexical Definition

A dictionary definition of entrapment infers a subjective feeling that all escape routes are blocked [13] and a strong desire to escape from difficult situations such as stressful events and hardships due to unexpected situations [13]. The sense of entrapment can be divided into sub-concepts of inner and outer entrapment, recognizing that inner entrapment is bound by one’s inner thoughts and feelings, and outer entrapment is bound by external circumstances [13]. In addition, entrapment is perceived to be blocked by a defense mechanism to avoid stress or escape from a situation that can cause stress [25], which leads to psychological symptoms such as depression.

Due to the COVID-19 situation, people face inequality and injustice from coercive control in particulars of daily activities [26]. People experience guilt, fear, and stress while pushing for social contact alongside outings if the feeling of entrapment becomes more substantial due to the pandemic [27]. Furthermore, its long-term continuation severely affects an individual physically and mentally and society as a whole [27]. The environment during COVID-19 is uncontrollable and unavoidable and involves a subjective desire to escape from the currently oppressed situation [28]. This unpredictability is an important issue that can lead to suicidal ideation [29]. Feelings of entrapment are subjective emotions that can arise from social coercive deterrence measures such as ‘social distancing’ and feeling no hope in a situation.

#### 3.1.2. Range of Use of the Concept

Looking at the use of the concept of entrapment in other disciplines, entrapment in psychiatry is used as a crisis intervention study to treat survivors of catastrophic situations [30]. Here, entrapment is defined as the inability to control, separate, and accept an individual’s perception of their situation [30]. It is also expressed as “arrested escape”, feeling highly stressed by a desire to escape but finding the escape route blocked due to high barriers [30]. In particular, it appears due to various reasons like depression, post-traumatic stress disorder, and anxiety and may ultimately lead to a suicidal crisis [30]. In neurology, the concept of entrapment is seen as “nerve compression” [31]. Due to the compression of the nerve, there is a lack of proper blood supply, symptoms such as peripheral neuropathy and ischemic edema appear, and nerve conduction is slowed or blocked [31]. In jurisprudence, entrapment is seen as a “trap” [32]. This generally refers to a pitfall that entices law-abiding people to commit crimes they would not otherwise have committed [32]. Entrapment in counseling psychology is expressed as a failure to control overwhelming and negative emotions and the inability to cope with specific situations due to increasing psychological problems adequately [33]. In nursing, entrapment usually appears in families caring for cancer patients. They experience a lot of stress and burden as they continuously care for cancer patients and solve various problems [34]. While caring for cancer patients, they do not have enough personal time for themselves, which increases the sense of bondage, lowering the health and quality of life of caregivers [34].

### 3.2. Tentative Criteria for and Attributes of Feelings of Entrapment

**Figure 3:**
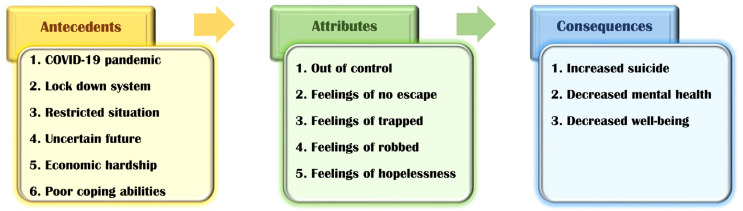
Concept diagram of “Feelings of entrapment”.

The following provisional criteria for attributes of feelings of entrapment during the COVID-19 pandemic were identified by reviewing various pieces of literature (Table 1, Figure 3).

(1) Out of control

The situation during the COVID-19 pandemic is out of control; an individual’s ability to control impulses and modify inappropriate emotions and thoughts [48,49] and the world is desperately exhausted [48]. Out of control is. Low self-control is also a significant risk factor in interpersonal relationships. Higher self-control leads to greater adaptability, less binge eating and alcohol abuse, improved interpersonal and interpersonal skills, stable attachment, and optimal emotional response [50]. In the COVID-19 context, people feel little control over this threat, and anxiety and depression symptoms can develop when they cannot adequately handle this feeling using their existing psychological resources [51,52,53]. In addition, trauma survivors report feeling entrapment and loss of control that have consequences for their mental health [54]. The situation is practically out of direct personal control. Therefore, a way to provide counseling services that can help individuals cope with the sense of loss of control is required. In addition, there is a need for institutional improvement measures that can be directed at the government level.

(2) Feelings of no escape

Entrapment is the feeling of being highly motivated to escape one’s situation but unable to [55], which is the case in the current pandemic [35,44]. It leads to feelings of defeat from no visible escape from the nefarious consequences of COVID-19 [35]. In particular, it adds further stress to those trying to cope with depression [44]. Therefore, positive psychological interventions will be needed to reduce the negative thinking that we cannot escape endlessly from the COVID-19 pandemic and reduce people’s mental health deterioration.

(3) Feelings of trapped

Feelings of entrapment denote feeling trapped [3,43,44] and are composed of two subtypes, internal entrapment and external entrapment [56]. Internal feelings of entrapment refer to being trapped by internal aspects such as one’s thoughts, and external refers to being trapped by the external environment [44]. These feelings of being trapped with no end in sight make one feel powerless for the future [44]. The lockdown imposed to contain the spread of COVID-19 has led to an economic crisis. Moreover, the constant need to be with a partner all day has led to abuse. The lockdown increased the risk of domestic violence and children witnessing parents fighting, leading to high psychological stress, the prevalence of suicide, and mental instability [57,58]. Therefore, to overcome feeling trapped and abuse caused by lockdown, counseling through video calls and healthy communication with surrounding people are needed.

(4) Feelings of robbed

COVID-19 pandemic is robbing families of the chance to say a final goodbye [59]. Accepting death without saying goodbye to the bereaved family is more difficult, adding psychological pain for those who have lost family and friends [59]. Moreover, peer support can be crucial for mothers and babies [60]. Due to the pandemic, health professionals cannot interact face-to-face with mothers; instead, they primarily contact by phone or virtual means [61]. Mothers with infants expressed that this may be necessary for practical support, especially first-time mothers. Moreover, online or phone support is helpful for emotional and social support [62]. Feeling robbed has been presented as an attribute of a sense of entrapment in the pandemic [43] and has led to despair [62]. Therefore, physical, online, and phone contact can help provide practical, emotional, and social support for those in need [62].

(5) Feelings of hopelessness

The continuation of the COVID-19 pandemic situation has led to the feeling of hopelessness [29,45,46]. Particularly, it arises from the feeling that all efforts aimed at constructive change in a patient’s recovery will be futile before an attempt is even made. It is important to identify factors affecting anxiety, despair, and individuals more psychologically affected during the COVID-19 pandemic [63,64]. Clinical psychologists often refer to depression as being characterized by self-defeat [13] (hopelessness and helplessness), leading to a state of “arrested flight” or feelings of entrapment [35]. Sustained hopelessness is positively correlated with demoralization and eventually influences suicidality and suicidal ideation [46]. Moreover, it may increase national anxiety and hopelessness in situations of uncertainty, such as the pandemic [64]. Therefore, in the current prolonged situation, it is necessary to prepare for prevention and treatment at the individual and national levels to not feel hopeless.

### 3.3. Construction of a Model Case of the Concept

The model case of a concept refers to a case that includes all the key attributes [22]. This study presents the model case based on the four primary attributes of a COVID-19 healthcare safety net. The attributes are indicated with their numbers in parentheses.

Stephanie, 29, is an ordinary office worker. She went camping with her friends and tested positive for COVID-19. The government directed her to be quarantined, moving her in quarantine at a COVID-19 hospital. She felt highly frustrated during this time. The isolation ward consisted of single rooms and required protective gear use. Outsiders’ access was strictly blocked (Figure 3, attributes3), and medical personnel were also required to wear protective gear (Figure 3, attributes3) when entering the building. All the examination equipment was brought into the room for proceeding, and there was no opportunity to leave the room (Figure 3, attributes2). In addition, it was aggravating not to contact family and friends because their cell phones were not allowed. After about a month of this condition, Stephanie felt trapped in a small single room since she could not go out (Figure 3, attributes2). She felt as if someone had forcibly locked her and was watching her (Figure 3, attributes4). She asked the medical staff to allow her for a walk in the hospital’s park (Figure 3, attributes4). However, the medical team explained why they could not risk the spread of the COVID-19 proton. Hence, she felt further trapped by the COVID-19 (Figure 3, attributes3), despite explaining that she could be discharged after a physician confirms a negative reaction for the infection by periodically performing a COVID-19 test. Afterward, Stephanie realized that she could not go out (Figure 3, attributes2) and fell into deep despair (Figure 3, attributes5).

### 3.4. Additional Cases of the Concept

#### 3.4.1. Borderline Case

A borderline case includes a few key attributes of the concept presented in the model case [22]. In this study, the borderline case addresses the issues of being out of control, feelings of no escape, and feelings of being trapped.

Jane, 70, is a kidney disease patient on hemodialysis. She is on dialysis on Mondays, Wednesdays, and Fridays and has a long history of fighting illness. She has been hospitalized for treatment for about a month, and due to COVID-19, is living with a caregiver without visiting her family. Jane received dialysis in the dialysis room and returned to her room, where she was informed of a confirmed COVID-19 case in the hospital. She was asked to conduct a COVID-19 test since she was exposed to the patient and could go to her room if the test came negative. Due to this, she suddenly felt that everything was blocked because she had to move from a single room to an isolation room suddenly (Figure 3, attributes3). However, knowing that the COVID-19 situation required cooperation, she knew isolation was inevitable (Figure 3, attributes1). In the quarantine room, she was provided a single room (Figure 3, attributes3). The room was with no music, television, or other entertainment resources. Soon, Jane felt trapped, under pressure to stay disengaged (Figure 3, attributes1), had difficulty breathing, and started acting out. However, the medical staff explained to her that the regulations prevented her from moving to the general hospital room. She also recognized that with cooperation through the quarantine, she could move to the general hospital room.

#### 3.4.2. Contrary Case

This type of case does not display attributes of the concept and presents attributes contrary to it [22]. Its presentation allows the attributes of the concept being used to be better understood and clarified. The following case did not include any attributes of feelings of entrapment during the COVID-19 pandemic, and thus, it can be considered a contrary one.

Rachel, in her 30s, is a lawyer. She goes to work every day at her office. Due to the pandemic, she has been working from home since last year. One day, while telecommuting, she was told that a staff member in her office had been positive for COVID-19. The given staff had to live in quarantine after leaving the hospital that manages COVID-19 cases, disinfecting their offices. However, since Rachel was working from home, she did not undergo a separate COVID-19 test. In addition, since she works from the comfort of her home, it makes her less frustrated than the employees who have to live in isolation. She can go return to work after the office is disinfected and received negative results from other employees’ COVID-19 tests.

#### 3.4.3. Related Case

A related case does not have the key attributes of the concept [22]. While there may be some similarities between the concepts, the differences in the attributes are analyzed, and thus, it has a different meaning in concept analysis.

Mike, in his 20s, is a nurse working at a university hospital. At the end of 2019, he applied for work in the COVID-19 isolation ward. He works in the isolation ward wearing protective gear and blocking contact with the outside world. While eating, he can only eat on the appropriate route because he could not bring food stepping outside. Moreover, he had to remove his protective gear when coming out of the isolation room and wear a new protective gear when stepping back into the room. Mike works in a blocked state but has to cope with it as a medical staff. It is challenging to live in isolation as medical staff and work hoping for the COVID-19 situation to end.

### 3.5. Identification of Antecedents and Consequences of Feelings of Entrapment

#### 3.5.1. Antecedents

Antecedents refer to additional conditions or events before the occurrence of the concept [22]. Based on the literature review, the following antecedents of feelings of entrapment during the COVID-19 pandemic were identified (see Table 1 for more details).

1. COVID-19 pandemic
2. Lockdown system
3. Restricted situation
4. Uncertain future
5. Economic hardship
6. Poor coping abilities

#### 3.5.2. Consequences

Consequences refer to additional conditions or events after the occurrence of the concept [22]. The following consequences of feelings of entrapment during the COVID-19 pandemic were identified.

1. Increased suicide
2. Decreased mental health
3. Decreased well-being

### 3.6. Empirical Criterion

Empirical Criterion is the final step in conceptual analysis to show that the properties of the concept exist in the actual field (Walker and Avant, 2005). Entrapment’s measurement tools are measured by Gilbert and Allan (1998) [13] and consist of two subscales, including internal and external entrapments. Internal entrapment relates to the concept of self-evasion, trying to escape from inner emotions and thoughts. External entrapment represents the situation or person in the external world that induces escape motivation. In this study, the empirical observance of feelings of entrapment during the COVID-19 pandemic revealed similar movements to control individual freedom and privacy and to prevent the spread of disease worldwide [65]. In the process, people were allowed to step out to buy groceries or enter under the system of each country by applying age restrictions [65]. Currently, lockdown restricts individuals to stay at home strictly, and those quarantined report a rise in suicide compared to others [66]. Individuals also feel isolated from the outside world [16], reported feeling trapped in their house, and expressed concerns about the prolonged pandemic [67].

## 4. Discussion

COVID-19 is on the rise worldwide and is an unprecedented problem. There is a need for mental health care, and adequate services must be provided for those at risk of and existing mental health problems [14]. The main adverse effects of the COVID-19 pandemic include anxiety, depression, self-harm, and suicide attempts due to increased social isolation and loneliness. It would be necessary to know about the possible factors [1]. Additionally, since individuals’ mental health after COVID-19 will vary with background and individual circumstances, preventing long-term disability and minimizing these sequelae requires expanding trauma-focused resources and expertise [68]. Moreover, there is a need to study which aspects of the COVID-19 pandemic contributed to the adverse mental health outcomes and the factors and actions that could protect them [3].

Isolation, loneliness, and feeling trapped have been identified to impact nearly half of individuals admitted to hospital for self-harm after lockdown restrictions were introduced [16]. This approach to emotions is becoming increasingly important in the face of the COVID-19 pandemic and related social contact measures [11]. O’Connor (2011), based on a suicidal motivation-will integration model, suggests that defeat and humiliation can lead to feelings of being trapped, leading to suicidal intentions and behaviors [69]. Therefore, this study aimed to reduce the suicide rate by reducing the feelings of entrapment that negatively affect mental health by identifying and preventing its factors.

This study used the concept analysis developed by Walker and Avant (walker) [22] and the ACE star model [24] to analyze the concept of the feelings of entrapment during the COVID-19 pandemic. Out of control, feelings of no escape, feelings of trapped, feelings of robbed, and feelings of hopelessness were identified as the attributes of this analysis.

First, we discuss “out of control”. During the COVID-19 pandemic, people experienced increased mental distress and were also expected to exercise high levels of self-control concerning personal and social health behaviors [49]. Self-control acts as a buffer and allows one to better cope with the stresses resulting from COVID-19, and the general psychological distress decreases when one perceives they can exercise self-control [49]. In particular, the consequences of pitfalls at the international level may be more severe than those at the national, interpersonal, or interpersonal levels since the consequences of falling into a trap are likely to become out of control [69]. Thus, the situation must continue to avoid falling into the trap, but maintaining control requires a comprehensive strategy and informed and effective communication to create transparency [69]. Additionally, healthcare professionals need to be encouraged to exercise self-control to face individual existential questions and struggles [49].

Second, the feelings of no escape occur when we perceive no visible solution to escape the chronic stressful situation wherein the feeling of entrapment has progressed [70]. When people experience a severely stressful or threatening event, the fight-or-flight response is activated as a primary defense strategy [70]. In this case, the stress-coping method is used to adjust the relationship between feelings of entrapment and psychological well-being [70].

Third, the feelings of entrapment are shared in those who perceive that they lack resources, are primarily in chronic stressful events, or face difficulties in interpersonal and occupational relationships [28]. It is also observed in desperate and helpless people who express their state as a “black hole” and “blocked up and no way out” [28,70]. Decreased problem-solving skills and lack of social support lead to feelings of defeat and traps and increases suicidal thoughts and behaviors [71]. It is necessary to develop programs and skilled nursing strategies to prevent this [72].

Fourth, people feel robbed after losing several things due to the COVID-19 pandemic. The fear of not taking the virus home and self-isolation robs one of peace and intimacy with the loved one [73]. As such, measures for the grief of mental health due to COVID-19 are essential at a time when deaths continue to rise [74] and measures to meet daily deprivation of desires to prevent post-traumatic stress disorder and depression [73].

Fifth, we consider the feelings of homelessness and a worldwide epidemic such as COVID-19 that imposed strict quarantine and measures such as social distancing [46]. Strict restrictive situations have a severe impact on people’s mental and physical health [46]. The effects of economic and social turmoil during COVID-19 exacerbate hopelessness and suicidal impulses [3], especially entrapment, which is closely related and linked to suicide [29]. Among medical workers who play a critical role in this situation, nurses have higher levels of hospitality than other medical staff [64]. Most health care workers are at high risk and under intense pressure, including fear of infecting their families and others and increasing working hours, leading to feelings of anxiety, fear, hopelessness, and uncertainty [74,75]. Psychological and social intervention is needed for healthcare workers fighting against COVID-19 [75], and it is necessary to continuously monitor their healthcare and psychological well-being continuously [76].

In the COVID-19 paradigm, health professionals should use EBP to communicate optimal methods while focusing on population health, disease prevention, and adopting healthy lifestyles [20]. For this, the ACE star model based on the EBP served as an appropriate framework. It provided a foundation for establishing more explicit concepts through the entrapment experienced by COVID-19. Moreover, the steps in knowledge transformation provided a systematic framework for re-established concepts and were applied as opportunities for information [77]. Recognizing the importance of entrapment in the COVID-19 paradigm experienced globally and developing new knowledge resources to affect healthcare positively is necessary [20].

In Korea, as vaccinations began, many elderly facilities developed COVID-19 virus, and breakthrough infections occurred after vaccination [78]. Korea is strengthening social distancing to prevent COVID-19 [78]. They experience great economic losses due to containment such as upgraded social distancing, and the government recommends the use of masks even more [78]. In addition, due to the COVID-19 pandemic, Corona Blue happens, coined the term [79]. This indicates the frustration caused by the inability to engage in external activities due to COVID-19, anxiety that one may be infected, and lethargy caused by activity constraints [79]. In Korea, due to Corona Blue, people experience depression, self-harm, and psychiatric counseling, and especially as childcare hours for children at home increase, isolation, and depression among young people in their 20s and 30s are getting worse, leading to self-harm [79]. The Korean government has set up and operated a psychological support team within the Central Disaster and Safety Countermeasure Headquarters for many people suffering from Corona Blue, and psychological counseling programs for confirmed patients and their families are also underway at the National Trauma Center and National Psychiatric Center [79].

The COVID-19 pandemic is expected to continue for an extended period [80]. Due to the COVID-19 pandemics, the world has experienced a social network shutdown by implementing regional lockdowns, home isolation, and social distance [81]. Continued social isolation can lead to changes in health behavior, and smaller social networks with less medical support can further exacerbate this situation [82]. In particular, students have lost the opportunity to study in school, parents have difficulty creating a systematic environment for their children [83], and the elderly are more vulnerable to social isolation because of their high dependence on community services [4]. Adhering to all precautions, such as hand hygiene and proper wearing of masks, also acts as a stressor in the community, causing panic disorders, stress, anxiety, and economic fear [83]. Community members experience mental health problems due to lack of physical interaction, feeling entrapped in the situation [83].

The community should provide mental health services to those feeling entrapped by COVID-19 [84]. Psychologists should be employed in each region to provide professional psychological crises intervention to patients, families, and healthcare workers. Volunteers and psychological crisis intervention hotlines should be set up with community-based organizations to provide psychological support for healthcare workers at the forefront of social isolation and COVID-19 [84]. In addition, the importance of family ties must be emphasized, spending quality time with them, and eating healthy and exercising, based on the research that people with higher family ties are less likely to complain of mental problems [85].

Mental health problems play an important role in nursing in preventing diseases and improving the health of the people [86]. In particular, the risk of mental health problems in people around the world is increasing as the COVID-19 pandemic experiences entrapment such as lockdowns and physical distancing [87]. From a nursing perspective, remote services or monitoring should be provided for isolated patients or vulnerable groups for feelings of entertainment during the COVID-19 pandemics [88,89], enabling virtual mental health services and programs for them [84]. Quality mental health services such as introducing a helpline number for those who feel trapped must be provided to deal with social isolation [83]. In addition, mental health assessment tools should be developed along with necessary interventions at national and local levels to alleviate the long-term mental health of community members [83].

Among the antecedents identified in this study, in particular, the lockdown system and restricted situation are the preceding factors that appear as the COVID-19 pandemic continues. In addition, the attributes suggested through this study can be applied when composing a toll to measure the degree of restrain in the future. The development of these intervention programs and measurement tools will help decrease suicide and increase mental health and well-being.

The study presents a conceptual analysis of entrapment that can be felt most significantly in the context of COVID-19. Our concept analysis is, however, subject to some limitations. We included studies with many different types to obtain the contents for antecedents, attributes, and consequences of feelings of entrapment during the COVID-19 pandemic. Therefore, we could not proceed with the risk of bias. In addition, we found the various antecedent factors for feelings of entrapment, but it was difficult to find various antecedent factors for the attribute part. Therefore, as the COVID-19 situation continues, it is expected that studies including the attributes of feelings of entrapment will continue. This study proposes:

1. Further research from a nursing perspective, including the nature of entrapment, is required based on the results shown in this chapter.

2. It is necessary to establish an institutional mechanism to improve feelings of entrapment through the inter-complementation of social, economic, and health care at a national and community level.

3. It is necessary to develop a program to alleviate the feelings of entrapment arising from the pandemic.

## 5. Conclusions

This study is a conceptual analysis study that identifies the meaning and properties of feelings of entrapment during the COVID-19 pandemic by Walker and Avant (2005) [22]. Experiencing entrapment during the COVID-19 pandemic has confirmed an increase in suicide, and that with deteriorating mental health, the well-being decreases. The leading factors found in this study were the lockdown system, restricted situations, unaccredited future, economic hardship, and poor coupling abilities caused by the COVID-19 pandemic. This led to feelings of no escape and being treaded. Although the global response to the COVID-19 pandemic is expected, it is only getting worse with the continuation of the COVID-19 pandemic. Thus, this study also promotes well-being by accurately recognizing and understanding the concept of entrapment experienced in the pandemic.

Therefore, this study suggests developing a program that can improve the feelings of entrapment based on the findings, and further research will need to identify the nature of entrapment in the COVID-19 pandemic distinctly.

## Figures and Tables

**Table 1:** Antecedents, attributes and consequences of feelings of entrapment.

Dimension	Sub-Dimension	Key Findings in Reviewed Literature
Antecedents	COVID-19 pandemic	
	Lockdown system	Lockdown system or situation during the COVID-19 [3,6,18,26,29,35,36,37,38,39,40,41], impairment to social and occupational functioning [9]
	Restricted situation	Social consequences of COVID-19 [3], restrictions [35], physical distancing and self-isolation [36], isolation [29,42], reduced social opportunities [42], social restriction [38], isolation and loneliness/reduced contact with key individuals/living alone/cessation or reduction of service (including absence of face to face support) [16], limited/restricted [43], social distancing [6,18,29,40,43], losing direct social and physical contact [9], restricted in a situation [44]
	Uncertain future	Uncertainty or concerns about the future [3], uncertainty [42], uncertain future [45], no end in sight asks them to feel helpless at least for the foreseeable future [44]
	Economic hardship	Economic consequences of COVID-19 [3], loss of jobs occupying the premium position [37], economic hardship [37,42], temporary or permanently lost job and income [43], economic recession [40], financial stress [29]
	Poor coping abilities	Stress of many perfectionists who are trying to cope with the pandemic [44], pooper coping abilities [29]
Attributes	Out of control	Out of control [43]
	Feelings of no escape	Cannot escape [35], feelings of no escape [44]
	Feelings of trapped	Feelings of trapped [3,43], trapped in a situation [44]
	Feelings of robbed	Feelings of robbed [43],
	Feelings of hopelessness	Hopelessness [29,45,46],
Consequences	Increased suicide	Suicide ideation [3,6], self-harm [15,16], risk factors of suicide [9,14,15,18], suicide [6,29,35,37,42,44], suicidal intent and behavior [47], suicidality [46], suicidal actions and ambivalent to living or dying [6]
	Decreased mental health	Decreased mental health problem [3,16,29,44], feeling isolated and the specific mirroring of abuse [38], foster neglect and abuse [40]
	Decreased well-being	Decreased well-being [3,29], negative wellbeing [36], affect their care and wellbeing [40]

## Data Availability

Not applicable.
